# RETRACTION NOTE TO: Effect of Intra Uterine Granulocyte Colony Stimulating Factor vs. Human Chorionic Gonadotropin at Ovum Pick-Up Day on Pregnancy Rate in IVF/ICSI Cases With Recurrent Implantation Failure

**DOI:** 10.5935/1518-0557.20240059

**Published:** 2024

**Authors:** Haitham Torky, El-Sayed El-Desouky, Ashraf El-Baz, Rania Aly, Osama El-Taher, Atef Shata, Ahmed Hussein, Heba Marie, Osama Deif, Ahmed Eldemery, Ashraf Abo-Louz

**Affiliations:** 1 Department of Obstetrics & Gynecology - October 6th University, Giza, Egypt; 2 Department of Obstetrics & Gynecology - Al-Azhar University, Cairo, Egypt; 3 Department of Obstetrics & Gynecology - Al-Galaa Teaching Hospital, Cairo, Egypt; 4 Department of Obstetrics & Gynecology - Matareya Educational Hospital, Cairo, Egypt; 5 Department of Obstetrics & Gynecology - Cairo University, Cairo, Egypt; 6 Department of Biochemistry - October 6^th^ University, Giza, Egypt

The Editorial Board has retracted this article. After publication, concerns were raised about this study’s reported data. We reached out to the authors to request their raw data, but they could not provide. Therefore, the Editorial Board no longer has confidence in the results presented.

